# Gaps in secondary prevention among stroke survivors in rural Gadchiroli, India: a community-based cross-sectional study

**DOI:** 10.12688/wellcomeopenres.16377.1

**Published:** 2020-11-03

**Authors:** Yogeshwar Kalkonde, Sunil Jadhao, Mahesh Deshmukh, Shoummo Sen Gupta, Abhay Bang

**Affiliations:** 1 Society for Education, Action and Research in Community Health (SEARCH), Gadchiroli, Maharashtra, 442605, India

**Keywords:** secondary prevention, stroke, rural, India, community-based, survey

## Abstract

**Background**: With epidemiological transition, stroke has emerged as a public health priority in rural India. However, population-level information on secondary prevention of stroke from rural areas of India and other low- and middle-income countries remains exceedingly rare.

**Methods**: In a cross-sectional community-based survey, trained surveyors screened a well-defined population of 74,095 individuals living in 64 villages in Gadchiroli district of India for symptoms of stroke. A trained physician evaluated screen positive patients, diagnosed stroke, measured blood pressure and collected information on prior diagnosis of risk factors and current use of medications using a structured questionnaire.

**Results**: A total of 265 stroke survivors were identified. Prior diagnosis of hypertension was made in 57.4%, diabetes in 9.8%, hyperlipidaemia in 0.4%, ischaemic heart disease in 1.5%. and atrial fibrillation in 1.1%. Blood pressure was uncontrolled (>140/90) in 46% of stroke survivors. Among men 71.2% used tobacco and 30% used alcohol, while among women 38.2% used tobacco and none used alcohol. Only 40.8% of stroke survivors were receiving antihypertensive medications, while 10.6% were on antiplatelet agents and 4.9% were on statins. In a multivariate analysis, age <50 years (OR 0.2, 95% CI 0.1-0.5), male sex (OR 0.2, 95% CI 0.2-0.8) and lower economic status (no assets vs four assets; OR 0.3, 95% CI 0.1-0.9) were associated with lower odds of receiving medications for secondary prevention of stroke.

**Conclusions**: There were significant gaps in secondary prevention of stroke in rural Gadchiroli. Healthcare programmes for secondary prevention of stroke in rural areas will have to ensure that blood pressure is adequately controlled, alcohol and tobacco cessation is promoted and special attention is paid to those who are younger, men and economically weaker.

## Introduction

Stroke is the third leading cause of death globally ^1^. The burden of stroke has now shifted from high income countries (HICs) to low- and middle-income countries (LMICs) like India and 75% of all stroke deaths now occur in these countries ^2^. In India close to 700,000 patients died due to stroke in 2016 ^3^. Recent studies suggest that stroke has also emerged as a public health problem in rural India where two thirds of India’s and about 12% of world’s population lives ^4– 7^. In a study from central India, stroke was the leading cause of death in a rural community ^4^. Prevalence and mortality rates and overall disease burden due to stroke also remain high in rural India ^3, 5, 6, 8^. These data indicate that strategies are urgently needed to reduce the burden of stroke in rural India where healthcare services are often inadequate.

Acute stroke care in the form of thrombolytic therapy and stroke units can improve outcomes after stroke ^9, 10^ but such care is often not available in rural India ^11^. Therefore, primary and secondary prevention of stroke becomes an important strategy to reduce preventable mortality and disability due to stroke ^12^. Among primary and secondary prevention, secondary prevention could be relatively easier to achieve from health systems perspective given that patients visit a healthcare facility after acute stroke and the motivation to take medicines for secondary prevention could be higher. However, the status of secondary prevention among stroke survivors in rural India remains largely unknown.

In this study, we assessed the status of secondary prevention among stroke survivors by evaluating prior diagnosis of risk factors, current use of tobacco and alcohol, blood pressure control and use of medications for secondary prevention of stroke in a rural area of Gadchiroli district in central India.

## Methods

### Ethical statement

The cluster randomized controlled trial under which the study was nested was approved by the institutional ethical committee of SEARCH and the trial is enrolled in the Clinical Trials Registry of India ( CTRI/2015/12/006424, 08/12/2015). During the screening survey, verbal consent was obtained from individuals, while written informed consent in the local language was obtained from patients by the physician before collecting information and conducting clinical examination. Consent was obtained for participation in the study and use of data without revealing personal details. Participants were also informed about their freedom to quit at any stage of data collection. Confidentiality of all information obtained was maintained. The procedures followed were in accordance with the Helsinki Declaration of 1975, as revised in 2000.

### Study area

**Figure 1.**
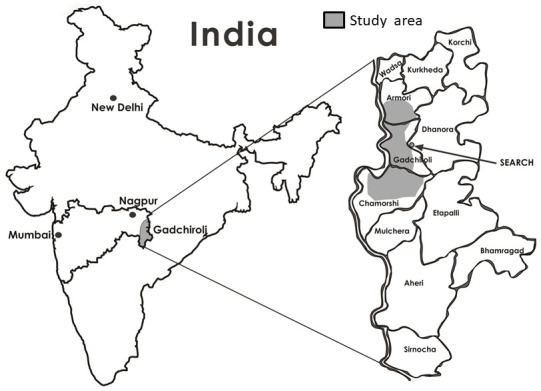
Study area.

This community-based cross-sectional survey was conducted in Gadchiroli district in central India ( Figure 1) which has a total population of 1,072,942 as per the Indian National Census conducted in 2011 ^3, 13^.

Within this district, 93% of the population lives in rural areas and 38.2% of the population is tribal. The literacy rate of the district is 66.03%. Subsistence farming and farm-labour are the main occupations ^14^.

Healthcare in the district is provided through the government healthcare system, private practitioners and a few voluntary organizations. There is only one neurologist (YK) and one computerized tomography (CT) scanner in the entire district and facilities to give intravenous tissue plasminogen activator (t-PA) after acute stroke are not available. A large number of informal or unskilled providers also treat various symptoms and are preferred by villagers given their easy availability and accessibility.

Society for Education, Action and Research in Community Health (SEARCH) is a non-governmental organization working in Gadchiroli district since 1986. It has a demographic surveillance system in 86 villages distributed in three clusters of the district: Gadchiroli, Armori and Chamorshi ( Figure 1). SEARCH has a population register in these villages which is updated yearly and all births and deaths are recorded.

### Sample size

The present study was nested in a cluster randomised controlled trial to assess the effect of a community-based intervention to reduce stroke deaths in rural Gadchiroli and was a part of a baseline assessment of risk factors and secondary prevention of stroke among stroke survivors ^15^. The trial was conducted in 64 villages selected randomly from 86 villages in the service area of SEARCH in Gadchiroli district. Out of these 64 villages, 32 villages each were randomised to the control and the intervention arm. Individuals were eligible to participate in the study if they were residents of these 64 villages and were prepared to give consent.

### Data collection

The details of the data collection method are described previously ^5^. Briefly, stroke patients were identified in the community by a three-stage cross-sectional survey. In the first phase, trained community health workers of SEARCH conducted a house-to-house survey from March 2016 to May 2016 using a validated questionnaire to screen the population for symptoms of stroke ^5^. The questionnaire inquired about anyone in the family ever having: a) weakness on one side of the body, b) numbness on one side of the body, c) weakness of face, or d) slurring of speech. Stroke was suspected if any one or more of these symptoms were acute in onset and lasted >24 hours. The questionnaire was evaluated locally in an earlier study and had sensitivity of 85% and specificity of 99% for diagnosing stroke ^5^. In the second phase of the study, patients suspected of having stroke were evaluated by a trained physician by making home visits from September 2016 to October 2017. Stroke was defined according to the World Health Organization’s clinical definition as a focal (or at times global) neurological impairment of sudden onset, lasting >24 hours (or leading to death), and of presumed vascular origin ^16^. The physician examined documents available with the patients to collect information on prior medical investigations and diagnoses of risk factors for stroke, inquired about current medication use, measured blood pressure and conducted clinical examination to confirm the diagnosis of stroke. Hypertension was defined as blood pressure >140/90 mm Hg. The information was collected using a structured questionnaire ^17^.

In the third phase of the study, patients whose diagnosis of stroke was unclear were evaluated by an external neurologist not associated with the trial from August 2017 to October 2017 to confirm the diagnosis of stroke.

### Statistical analysis

The information about the population in the 64 villages was obtained from the population register of SEARCH. We analysed group mean differences using the Student’s t-test and differences in percentages using the chi-square test. Logistic regression was used to examine the factors associated with use of any medication for secondary prevention of stroke with age, sex, education and economic status as independent variables. Data were analyzed using statistical software Stata version 10 (College Station, TX, USA). We followed STROBE guidelines for observational studies to report our findings.

## Results

**Figure 2.**
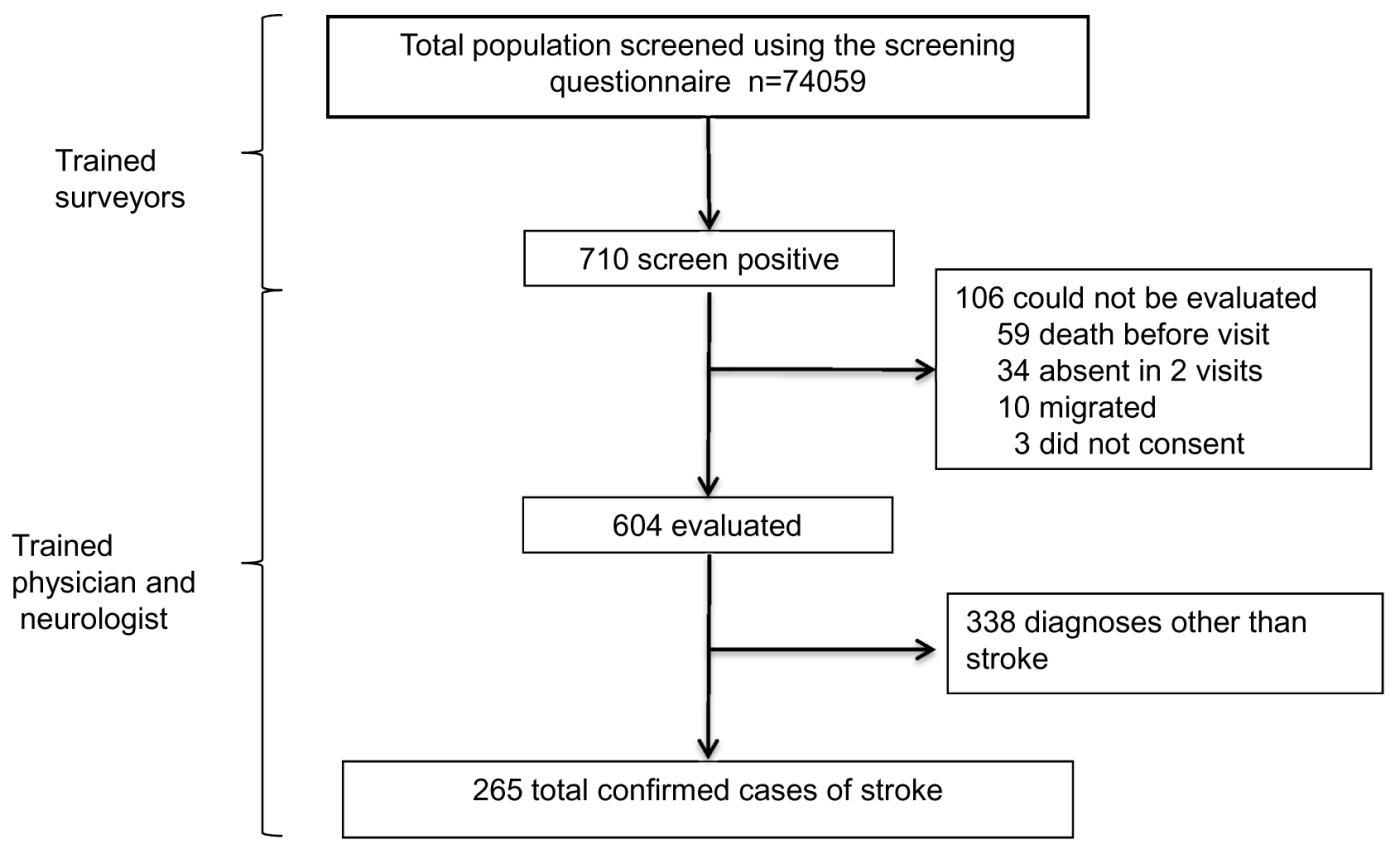
Flow diagram of the study scheme.

A total of 265 stroke survivors were identified after screening 74,095 individuals living in 64 villages ( Figure 2). Out of these, 102 (38.5%) were women and 163 (61.5%) were men ^17^. The demographic features of the stroke patients are shown in Table 1. The mean age of the patients was 61.9 years. The majority of the patients were illiterate with a higher number of women being illiterate than men (85.3% vs 33.1%, p<0.001). Regarding occupation, 47.2% were farmers and farm labourers, 35.4% were retired or not working and 14.7% were home makers. Household assets as an indicator of socioeconomic status are as shown in Table 1.

**Table 1.** Demographic characteristics of the study population.

	Women (n=102)	Men (n=163)	Total (n=265)
**%**	38.5	61.5	100
**Mean age in** **years (SD)**	61.8 (14.5)	61.9 (13.2)	61.9 (13.7)
**Education**			
Illiterate	87 (85.3)	54 (33.1)	141 (53.2)
Literate without schooling	1 (0.9)	1 (0.6)	2 (0.8)
Primary	6 (5.9)	61 (37.4)	67 (25.3)
Secondary and higher	8 (7.8)	47 (28.8)	55 (20.6)
**Occupation**			
Farming and labour	33 (32.4)	92 (56.4)	125 (47.2)
Home maker	39 (38.2)	0 (0.0)	39 (14.7)
Retired or not working due to other reasons	30 (29.4)	64 (39.2)	94 (35.4)
Service/business	0 (0.0)	7 (4.3)	7 (2.7)
**Household assets**			
Own farm land	88 (86.3)	139 (85.3)	227 (85.7)
Mobile phone	62 (60.8)	115 (70.6)	177 (66.8)
Television	47 (46.1)	87 (53.4)	134 (50.6)
Motorcycle	14 (13.7)	38 (23.3)	52 (19.6)

Among risk factors for stroke, hypertension was previously diagnosed in 152 (57.4%) and diabetes in 26 (9.8%). Very few patients were diagnosed with hyperlipidaemia (0.4%), ischaemic heart disease (1.5%) and atrial fibrillation (1.1%) ( Table 2).

**Table 2.** Risk factors diagnosed among patients with stroke.

Risk factors	Women (n=102)	Men (n=163)	Total (n=265)
Prior diagnosis of hypertension	67 (65.7)	85 (52.1)	152 (57.4)
Prior diagnosis of diabetes	8 (7.8)	18 (11)	26 (9.8)
Prior diagnosis of hyperlipidaemia	0 (0.0)	1 (0.6)	1 (0.4)
Prior diagnosis of ischaemic heart disease	2 (2.0)	2 (1.2)	4(1.5)
Prior diagnosis of atrial fibrillation	2 (2.0)	1 (0.6)	3 (1.1)
Current tobacco use	39 (38.2) *	116 (71.2)	155 (58.5)
Current smokeless tobacco use	39 (100)	108 (93.1)	147 (94.8)
Current alcohol use	0 (0) *	49 (30)	49 (18.5)

*p<0.05 for difference between women and men.

Among 152 patients with previous diagnosis of hypertension, blood pressure was uncontrolled in 84 (55.3%) patients. Out of 113 stroke patients without prior diagnosis of hypertension, 38 (33.6%) were found to be hypertensive during evaluation. Collectively, 190 (71.7%) patients had prior diagnosis of hypertension or hypertension at the rime of evaluation. Blood pressure was uncontrolled (>140/90) in 122 (46%) stroke patients [52 (50.9%) women vs 70 (42.9%) men, p=0.2].

Close to three quarters of men (71.7%) and more than a third of women (38.2%) were using tobacco, mostly smokeless tobacco. About one third (30%) of male stroke survivors were consuming alcohol ( Table 2).

Although prior diagnosis of hypertension was made in 152 (57.5%) stroke survivors, only 108 (40.8%) were receiving any antihypertensive medications, with significantly more women (51%) receiving the treatment than men (34.4%, p=0.007) ( Table 3). Only 10.6% of patients were receiving antiplatelet agents and even less (4.9%) were receiving a statin. Together, 117 (44.2%) stroke patients were receiving either an antihypertensive, antiplatelet or statin. In multivariate analysis, age <50 years (OR 0.2, 95% CI 0.1-0.5), male sex (OR 0.2, 95% CI 02-0.8) and lower economic status (no assets vs four assets; OR 0.3, 95% CI 0.1-0.9) were associated with lower odds of being on an antihypertensive, an antiplatelet agent or a statin ( Table 4).

**Table 3.** Medications used for risk factor control among stroke patients.

Medication	Women (n=102)	Men (n=163)	Total (n=265)
Any antihypertensive drug	52 (51) *	56 (34.4)	108 (40.8)
Any antiplatelet medication	9 (8.8)	19 (11.7)	28 (10.6)
Any statin	5 (4.9)	8(4.9)	13 (4.9)
Any antihypertensive/ antiplatelet medications or statin	55 (53.9) *	62 (38)	117 (44.2)

*p<0.05 for difference between women and men.

**Table 4.** Predictors of use of any medication for secondary prevention of stroke.

	n	Taking medicines for secondary prevention of stroke	OR (95 %CI)
**Age**			
>50 years	223	110	Reference
≤ 50 years	42	7	0.2(0.1-0.5)
**Sex**			
Women	102	55	Reference
Men	163	62	0.4 (0.2-0.8)
**Education**			
Literate	124	51	Reference
Illiterate	141	66	0.8(0.4-1.5)
**Number of** **household** **assets**			
4	47	30	Reference
3	68	29	0.4 (0.2-0.9)
2	67	25	0.4 (0.2-0.9)
1	64	26	0.4 (0.2-0.9)
0	19	7	0.3(0.1,0.9)

## Discussion

We found a significant gap in secondary prevention of stroke in this rural community despite stroke being a leading cause of death. Close to two thirds of stroke patients were diagnosed with hypertension, about one in twelve patients were diagnosed with diabetes, while few were diagnosed with hyperlipidaemia, heart disease or atrial fibrillation. Less than half of the patients were using antihypertensives and 10% or less patients were on antiplatelet medications and statins. Blood pressure was uncontrolled in close to half of all patients. To our knowledge, this is the first community-based study to evaluate use of medicines for secondary prevention of stroke in a rural community in India. It provides important insights regarding challenges to stroke prevention in a rural region of India where stroke has emerged as a public health priority.

Healthcare for stroke patients remains a challenge in rural India due to difficulties in accessing preventive care for non-communicable diseases. In our study, 57.4% of stroke patients were diagnosed with hypertension. This is an encouraging finding given that the study was conducted in one of the most underdeveloped districts of India. In previous population-based studies on stroke from relatively affluent rural areas of India, close to 90% of stroke patients had hypertension ^18, 19^ while in studies from rural Bangladesh and South Africa, 67.3% and 71% patients had hypertension, respectively ^20, 21^. Diagnosis of diabetes, hyperlipidaemia and ischaemic heart disease was infrequent in our study compared to reports from other community-based studies in rural India, where close to 50% patients had diabetes, 25% patients had hyperlipidaemia and 5-10% patients had atrial fibrillation ^18, 19^. However, these studies were conducted in academic medical centres and thus the likelihood of diagnosis of these risk factors would be high. The lower rates of diagnosis of risk factors in our study could be due to lack of access to healthcare in this district or that the study population is in an earlier phase of epidemiological transition where risk factors such as diabetes and hyperlipidaemia have not yet become common ^22^.

In resource poor settings where acute care for stroke is not easily available, secondary prevention of stroke becomes important to avoid preventable mortality. Longitudinal cohort studies have shown that the risk of cardiovascular events and death remains high for at least 10 years following stroke or transient ischaemic attacks ^23, 24^. The annual risk of vascular events was 2–5% in these studies ^23, 24^ and the 10-year risk of death was 43% ^24^. However, secondary prevention of stroke remains inadequate worldwide ^22^. In the Agincourt cohort in rural South Africa, 71% of stroke survivors had hypertension but only 8% were taking antihypertensive treatment ^21^. The PURE study assessed the use of medicines for secondary prevention of cardiovascular diseases in multiple countries. In this study, among patients with stroke in rural areas of LMICs, 33.6% were on blood-pressure lowering drugs, 27.3% were on antiplatelet agents and 2.4% were on statins ^22^. Comparatively, in our study, a somewhat higher percentage of stroke patients were on antihypertensive therapy (40.8%) and statins (4.9%) but a lower percentage were on antiplatelet agents (10.6%). However, it needs to be noted that the PURE study was conducted from 2003–2009 while our study was conducted in 2016 so it would be expected that a higher number of patients would be receiving these treatments. The pattern of relatively higher use of blood pressure lowering agents and lesser use of antiplatelet agents and statins that was observed in the PURE study was also found in our study. Given that close to 65% of strokes in rural areas of India are ischaemic ^19^, use of antiplatelet agents and statins in 10% or less stroke patients indicates a significant gap in the secondary prevention of stroke.

There are several barriers to use of medicines for secondary prevention of stroke in rural areas of LMICs. Availability and affordability of medicines for prevention of stroke are important barriers ^25^. A clinic-based prospective observational study from eastern India found that close to 35% stroke patients discontinued secondary preventive treatment of stroke over a two-year follow up period ^26^. The factors associated with discontinuation of treatment were lower socioeconomic and educational status, lower awareness, haemorrhagic stroke, higher daily cost of treatment and longer distance to the clinic ^26^. In our study too, lower socioeconomic status was associated with lower likelihood of use of medicines for secondary prevention of stroke. In addition, lack of availability of laboratory and imaging services in the district, low awareness among primary care physicians and reliance on informal providers could have contributed to the gap in the secondary prevention of stroke in our study. We are testing a community-based healthcare intervention to address these barriers ^15^.

Among stroke patients, significantly more women were receiving antihypertensive treatment compared to men (51 % vs 34.4%, p<0.05) and the same was true for being on an antihypertensive, antiplatelet treatment or statins (53.9% vs 38%, p<0.05). These findings are in agreement with previous studies on hypertension where more women were taking antihypertensive medications than men ^27– 29^. The potential reasons for this difference could be increased contact of women with the healthcare system during pregnancy ^27^ and more concern about health among women leading to a higher motivation to take medicines regularly.

Hypertension is the most important risk factor for stroke ^30^. Despite availability of medicines to control blood pressure, blood pressure control often remains inadequate ^27^. As far as blood pressure control among stroke survivors is concerned, hardly any population-level information is available from LMICs ^31^. In our study blood pressure remained uncontrolled in close to half of the stroke patients. This indicates that in addition to making medications available, there is a need to have adequate follow up to ensure that blood pressure is adequately controlled among stroke survivors who have hypertension.

In order to counter the rising burden of chronic non-communicable diseases, the Government of India has launched the National Programme for Prevention and Control of Cancer, Diabetes, Cardiovascular Diseases and Stroke (NPCDCS) ^17^, which is one of the largest programmes for prevention and control of chronic diseases in the world. It provides medications free of charge to patients at primary health centres. While this programme actively screens for hypertension, diabetes and cancers it is not currently screening for patients with stroke for secondary preventive treatment. Our findings suggest there is a need to actively seek patients with stroke in rural communities and start such patients on medications to prevent recurrence of cardiovascular diseases and death. As the medications are provided free in this programme, it helps to address the barrier of affordability. Providing preventive treatment at village level under this programme could help improve medication compliance and continuity. Our findings of lower use of stroke prevention medicines among those <50 years, men and those with lower socioeconomic status highlights that such patients would need special attention under the NPCDCS programme in rural areas.

Tobacco and alcohol are important risk factors for stroke ^30^. In our study, close to 70% of men and 40% women stroke patients were using tobacco, mostly smokeless tobacco. Tobacco use was rare among stroke patients in a population-based study in a rural area of Northern India, where only 3% stroke patients were using tobacco ^19^. On the contrary, in a study from southern India and rural Bangladesh, close to 40% of stroke patients were using tobacco ^18, 20^. One in three men with stroke used alcohol in our study. These findings are similar to those from Northern India, where 20% stroke patients were using alcohol ^19^. Our findings indicate an urgent need to reduce tobacco and alcohol consumption among stroke survivors.

A major strength of our study is the population-based sample of stroke survivors from a well-defined population in a demographic surveillance site. There are also certain limitations. Stroke cases were diagnosed using validated clinical diagnostic criteria as brain imaging was not easily available and affordable in this area. Also, we only recorded healthcare seeking among stroke survivors and not among those who died due to stroke. Some of the screen positive individuals died before evaluation by the physician and it is possible that some of them had stroke and their information was lost. We used self-reported retrospective information regarding treatment received for stroke. However, this information reported by stroke survivors was verbally confirmed by caretakers and consistent with the pattern observed by SEARCH which has been working in this area for more than 30 years. Also, we could measure only blood pressure and blood tests were not conducted given the reluctance of people to give blood for testing. Furthermore, though our study was conducted in a rural area of India, access to healthcare and socioeconomic status could be variable in various rural regions of India. This will affect use of medicines for secondary prevention for stroke. However, given that secondary prevention of stroke remains suboptimal even in the developed countries ^22^, our findings are likely to be true in other rural regions of India as well.

## Conclusions

Secondary prevention of stroke was suboptimal in rural Gadchiroli. The use of medications to prevent stroke was low, close to half of the stroke patients had uncontrolled blood pressure and use of tobacco and alcohol was common. These findings, in the context of high prevalence and mortality due to stroke, highlight an urgent need to improve secondary prevention of stroke in rural India.

## Data availability

### Underlying data

Figshare: Gaps in secondary prevention among stroke survivors in rural Gadchiroli, India: a community-based cross-sectional study. https://doi.org/10.6084/m9.figshare.13134782.v1 ^17^.

This project contains the following underlying data:

- kalkonde_et_al_data_secondary_prevention.xlsx (Data dictionary with demographic and clinical data)

### Extended data

Figshare: Gaps in secondary prevention among stroke survivors in rural Gadchiroli, India: a community-based cross-sectional study. https://doi.org/10.6084/m9.figshare.13134782.v1 ^17^.

This project contains the following underlying data:

- Kalkonde_et_al_physician_questionnaire.pdf (Physician questionnaire)

Data are available under the terms of the Creative Commons Attribution 4.0 International license (CC-BY 4.0).
