# Supplementary material for: Gaps in secondary prevention among stroke survivors in rural Gadchiroli, India: a community-based cross-sectional study
Source: Wellcome Open Res. 2020 Nov 3;5:263. [Version 1] doi: 10.12688/wellcomeopenres.16377.1 (PMC7721060; doi:10.12688/wellcomeopenres.16377.1)
Supplement: Peer review history

# Peer review: Reviewer response for version 1

**DOI:** 10.21956/wellcomeopenres.18013.r41250

**Published:** 2020-12-15

**Authors:** Giridhara R. Babu

**Affiliations:** 1 Department of Epidemiology, Indian Institute of Public Health, Public Health Foundation of India, Bengaluru, Karnataka, India

This is a very good contribution to the prevention efforts of NCDs in India. The study outlines the significant gaps in secondary prevention of stroke in rural Gadchiroli and offers solutions for future courses of action. It is unfortunate to see the focus only on curative aspects in public discourses and advocacies, often ignoring the prevention aspects. Therefore, this is a valuable contribution to strengthen public health programs in the country if the policymakers take cognisance of the importance of universal coverage of persons with high blood pressure. Similarly, the article underscores the importance of reduction of behavioural risk factors including alcohol and tobacco in rural areas. 

 Some of the minor review suggestions are provided below are adequately controlled, alcohol and tobacco cessation are promoted and special attention is paid to those who are younger, men, and economically weaker.

 Sl num

 Section

 Comments

**Abstract:**

1. Aim of the study can be mentioned in the abstract.
2. The role of surveyor and physicians are not clear. Does the surveyor refer the screened individuals to physicians or it was a house visit? Physician administered the questionnaire or survey staff? What are the risk factors? Please explain.

**Introduction:**

1. State the aim and objective of the study.

**Methods:**

1. Please provide the details about consent to participation and ethical consent?
2. Please define the term stroke survivor in the methods or eligible population.
3. Inclusion and exclusion criteria can be mentioned.
4. Sample size: The trial was conducted in 64 villages, it will help if the sample size is provided in terms of the number of persons in the area (men/women).
5. Data Collection: The interval of 1st phase, 2nd phase, and 3rd phase of the study is more? (1st phase ends in May and the second phase starts from September).
  History on alarming symptoms was collected in the 1st phase of the survey, where the participants may require immediate referral based on the severity of signs or symptoms. How does this interval justify ethically?
6. Data collection: What is the intervention given to the intervention group and how does it differ from the control group?
7. Data collection: Please include the section of parameters assessed during the data collection such as risk factors and medication history.
8. Data collection: Please mention who has done the data collection and training on data collection.
9. Data collection: Please provide information on the tools are used to measure the stroke?
10. Data collection: Which medical investigations conducted?
11. Data collection: Which instrument is used to collect BP measures digital/manual? Details regarding calibration are needed
12. Statistical analysis: Expand abbreviations on first use STROBE expand.
13. The method section does not include information on diabetes, hyperlipidemia and IHD diagnosis.

**Results:**

1. Who are the eligible participants? Why is a community based survey conducted if the purpose is to interview stroke survivors?
2. Please include which criteria were followed for classifying blood pressure? Please include the citation.
3. Age group specified wrongly (>=50years) in the description and please mention statistical significance in terms of p-value.
4. Table: Please mention n (%) in table heading for table 1,2,3

**Ethical statement:**

1. Expand abbreviation for SEARCH 

Is the work clearly and accurately presented and does it cite the current literature?

Yes

If applicable, is the statistical analysis and its interpretation appropriate?

Yes

Are all the source data underlying the results available to ensure full reproducibility?

Yes

Is the study design appropriate and is the work technically sound?

Yes

Are the conclusions drawn adequately supported by the results?

Yes

Are sufficient details of methods and analysis provided to allow replication by others?

Yes

Reviewer Expertise:

Non communicable diseases

I confirm that I have read this submission and believe that I have an appropriate level of expertise to confirm that it is of an acceptable scientific standard.

# Peer review: Reviewer response for version 1

**DOI:** 10.21956/wellcomeopenres.18013.r41251

**Published:** 2020-12-04

**Authors:** Rohina Joshi

**Affiliations:** 1 Faculty of Medicine, University of New South Wales (UNSW Sydney), Sydney, Australia

This is a well written and much needed paper about the prevalence of stroke from a rural region of India. The methods and data analysis are well described. The results are presented in detail, and strengths and limitations are described well. I have the following questions/comments:

1. The CHWs conducted the survey in 2016 and the physician assessed the individuals suspected to have strokes in 2017 (1 year later), were incident cases added to the survey?
2. Also, the survey may have missed minor strokes/strokes that result in death which would lead to an undercount.
3. While the Authors have given some context re public health facilities, it would be useful for the reader to understand the available health care facilities. Does the local PHC pharmacy store essential medicines for hypertension/ DM/ high cholesterol? Is there a 104 service in the villages?
4. Please include the age group of the surveyed population (74,095). Did this include population above 30 years or 18 years old adults?
5. Minor typo in results - rime, instead of time.

Is the work clearly and accurately presented and does it cite the current literature?

Yes

If applicable, is the statistical analysis and its interpretation appropriate?

Yes

Are all the source data underlying the results available to ensure full reproducibility?

Yes

Is the study design appropriate and is the work technically sound?

Yes

Are the conclusions drawn adequately supported by the results?

Yes

Are sufficient details of methods and analysis provided to allow replication by others?

Yes

Reviewer Expertise:

Health Systems

I confirm that I have read this submission and believe that I have an appropriate level of expertise to confirm that it is of an acceptable scientific standard.

# Peer review: Reviewer response for version 1

**DOI:** 10.21956/wellcomeopenres.18013.r41252

**Published:** 2020-12-03

**Authors:** Mahesh Kate, Chetan Vekhande

**Affiliations:** 1 Division of Neurology, Medicine, University of Alberta, Edmonton, AB, Canada; 2 Alberta Health Services, Edmonton, AB, Canada

**Secondary Stroke Prevention in Rural and Tribal Region: When Treatment Gaps are as Wide as Valleys.**

 The authors should be commended for conducting an important study, which quantifies the unmet needs of secondary stroke prevention in rural and tribal regions.

 The authors conducted a survey in 64 villages with a total population of 74,095 people to identify people in a three-tiered manner. First, a community health worker went house to house with a validated questionnaire. Patients with suspected stroke were then seen by trained physicians by home visits to diagnose stroke. There was additional confirmation for some borderline cases by an external neurologist if required. A total of 265 stroke survivors were identified with a mean age of 62 years and 62% were male. In patients known to have high blood pressure >50% were uncontrolled. Current tobacco use was noted on 59%. Diabetes, dyslipidemia, and atrial fibrillation were less likely to be diagnosed. Interestingly more women were likely to be on medication compared to men.

 A similar study has been conducted in rural Punjab (Northwest region of India) in Ludhiana district in 164 villages with a population of 173, 852 ^1^. A total of 359 first-ever strokes were identified with a mean age of 62 years and 50% were male. The prevalence of hypertension was 89% and current tobacco use at 24%. A small percentage of patients were on medication and had controlled blood pressure. Half (50%) of the stroke patients visited unqualified medical practitioners for acute stroke care.

 Though pharmacological management is pivotal for secondary stroke prevention, non-pharmacological measures are important adjuncts as well. Few may argue that they are equally important. In the INTERSTROKE study lack of regular physical activity and diet poor in fruits and vegetables was associated with 58% of population attributable risk of stroke ^2^. In Ludhiana rural stroke Registry 17% of patients with first-ever stroke had a sedentary lifestyle and 59% had a high degree of unsaturated fat consumption. In the Gadchiroli population in the current study, 35% were retired and not working. Data about diet is not available. 

 Both studies (Gadchiroli and Ludhiana) highlight an important ‘valley-sized’ health care gap in stroke prevention in rural regions. According to the World Bank data (2019), 44% of the world population resides in rural regions this number in India is 60%. In the USA a total of 63% of areas with health care professional shortage were in rural regions (ruralhealthinfo.org). According to the Centre for Disease Dynamics, Economics and Policy (CDDEP) report in 2019 India has a shortage of 600,000 doctors and 2 million nurses. The shortage is magnified in rural and tribal regions.

 In addition to the barrier of poor access to health care professionals, the lack of trust in modern systems of health care is evident. This is partly due to costs of treatment and lack of communication between the healthcare providers and patients.

 Another important barrier for healthcare delivery and access is disability associated with stroke. We do not have information regarding the modified Rankin scale or some disability assessment measure from the current study. Depending on the premorbid status, stroke severity and associated comorbidities the disability is variable with >50% having some persistent deficits. 

 Novel policy intervention will be required to bridge these gaps. The authors are conducting a task-sharing trial with mobile outreach clinics and community health workers with the aim to prevent stroke-associated mortality ^3^. In Ludhiana Rural stroke Registry a trial to improve control of hypertension in stroke survivors with task shifting measures with ASHA (Accredited Social Health Activists) and remote physician was conducted recently ^4^. A total of 140 patients were randomised to ASHA assisted BP control or standard care available in the region. The authors noted a dramatic 28 mmHg systolic drop in blood pressure in the intervention arm, this was primarily due to the increase uptake in medication. This needs replication in other regions to see clinical effectiveness i.e. a decrease in stroke and mortality. This data will soon be available from the Gadchiroli intervention study.

 The healthcare disparity in rural regions poses a formidable challenge. It is important to identify and quantify them which the current study has done. Task sharing may be an effective tool to help us address it, but it remains to be proven in randomised control study.

Is the work clearly and accurately presented and does it cite the current literature?

Yes

If applicable, is the statistical analysis and its interpretation appropriate?

Yes

Are all the source data underlying the results available to ensure full reproducibility?

Yes

Is the study design appropriate and is the work technically sound?

Yes

Are the conclusions drawn adequately supported by the results?

Yes

Are sufficient details of methods and analysis provided to allow replication by others?

Yes

Reviewer Expertise:

Stroke, Clinical trials, Neurology, Patient education, secondary stroke prevention

We confirm that we have read this submission and believe that we have an appropriate level of expertise to confirm that it is of an acceptable scientific standard.

## References

[1] : Establishment of Acute Stroke Care Pathway and Stroke Surveillance System in Rural Northwest India Using Community Health Workers. *SSRN Electronic Journal*.2019; 10.2139/ssrn.3487748 10.2139/ssrn.3487748

[2] : Global and regional effects of potentially modifiable risk factors associated with acute stroke in 32 countries (INTERSTROKE): a case-control study. *The Lancet*.2016;388(10046) : 10.1016/S0140-6736(16)30506-2 761-775 10.1016/S0140-6736(16)30506-2 27431356

[3] : Effect of a community-based intervention for cardiovascular risk factor control on stroke mortality in rural Gadchiroli, India: study protocol for a cluster randomised controlled trial. *Trials*.2019;20(1) : 10.1186/s13063-019-3870-x 764 10.1186/s13063-019-3870-x 31870394PMC6929484

[4] : Accredited social health activist for vascular risk factor control in rural stroke patients (ASSIST) study. *Int. J. Stroke*.2020;15:115

# Peer review: Reviewer response for version 1

**DOI:** 10.21956/wellcomeopenres.18013.r41253

**Published:** 2020-12-01

**Authors:** Rita Krishnamurthi

**Affiliations:** 1 National Institute for Stroke and Applied Neurosciences, Faculty of Health and Environmental Studies, Auckland University of Technology, Auckland, New Zealand

This is a cross-sectional study of a subset of participants enrolled in a randomized controlled trial. The setting is rural central India. The parent study screened trial participants for stroke risk factors and stroke. The current study aimed to evaluate the status of stroke risk factors in the sample of participants diagnosed with stroke based on a 3-tier system of identification of stroke cases. The premise is that people at high risk of secondary stroke and the associated morbidity and mortality are more easily identifiable and able to be targeted for secondary prevention. The findings showed that just over 57% of the population were diagnosed with hypertension, but only about 41% were receiving any medications. Other risk factors such as diabetes and hyperlipidemia were diagnosed in a very small proportion of the sample. The overall findings suggest significant gaps in secondary prevention, particularly in younger men and those with lower socioeconomic status. These are important findings that, if acted upon, can lead to significant improvements in outcomes.

 The authors are to be commended on conducting this research in a highly vulnerable yet challenging community setting, where even the most basic level of healthcare is not easily available to large sections of the community. The mean age of stroke is relatively low (68 years) in this population.

 The work is well presented, clearly written, and cites the relevant literature. The study design is appropriate and acknowledges the limitations of the study settings, particularly in terms of stroke diagnosis. The authors provide adequate detail in the paper or via the references to their related work. The analysis is described clearly. The full data is not provided in this manuscript, but references provide links to associated data. I am assuming raw data may be requested.

 The Discussion offers sound and reasonable explanations for findings while describing limitations. The one missing element may be suggestions for the use of this data to make an impact on stroke burden.

 The authors also mention that this particular population may yet be in the early stages of epidemiological transition and may have a lower prevalence of diabetes and hyperlipidemia than the overall population of India. This may suggest targeted prevention strategies aimed predominantly at hypertension and alcohol use.

 I have the following queries:

- From the screening questionnaire, it is unclear if minor strokes were able to be picked up. Were vision problems and any cognitive deficits also screened for?
- From the parent paper (Ref #15) it is seen that participants were screened for stroke risk factors in the community. While screening for hypertension is simple, and stroke diagnoses were established with a questionnaire, it is not clear how the presence of diabetes was confirmed. Were further tests conducted by a physician after urine tests were done?
- How was the dispensation of, and compliance to prescribed medications tested?
- In terms of alcohol and tobacco as risk factors, could the authors clarify whether the type of alcohol used (e.g. homemade or potentially toxic) leading to worse outcomes in men? Similarly is there any data on the relative risk of smokeless tobacco versus tobacco smoking for stroke?  
- It's mentioned on Page 7 that 65% of strokes are ischemic. This is a lower proportion than would be expected (usually around 75-85%). Could the authors offer any explanation for this in this sample? What would the implications for secondary prevention be for 35% of people with haemorrhagic stroke? For example, would targeting alcohol use be more beneficial in this group.
- The authors point to encouraging government actions to target NCDs that could be a possible avenue to target stroke secondary prevention with the dispensation of free medications. What mechanisms are in place to allow the delivery of this programme to this population?
- Interestingly, it is mentioned that patients are often reluctant to give blood. Is there a role CHW could play in also addressing some of these issues, including low awareness which was also mentioned? For example what specific lifestyle risk factors for hypertension (e.g. high salt intake) could be targeted for prevention? 
- Minor typo Page 5 2nd line (rime instead of time)

Is the work clearly and accurately presented and does it cite the current literature?

Yes

If applicable, is the statistical analysis and its interpretation appropriate?

Yes

Are all the source data underlying the results available to ensure full reproducibility?

Partly

Is the study design appropriate and is the work technically sound?

Yes

Are the conclusions drawn adequately supported by the results?

Yes

Are sufficient details of methods and analysis provided to allow replication by others?

Yes

Reviewer Expertise:

Stroke epidemiology, stroke prevention

I confirm that I have read this submission and believe that I have an appropriate level of expertise to confirm that it is of an acceptable scientific standard.
